# It's in the bag: mobile containers in human evolution and child development

**DOI:** 10.1017/ehs.2020.47

**Published:** 2020-10-01

**Authors:** Thomas Suddendorf, Kelly Kirkland, Adam Bulley, Jonathan Redshaw, Michelle C. Langley

**Affiliations:** 1 Centre for Psychology and Evolution, School of Psychology, The University of Queensland, St Lucia 4072, Australia; 2 Department of Psychology, Harvard University, Cambridge, MA 02138, USA; 3 The University of Sydney, School of Psychology and Brain and Mind Centre, NSW 2050, Australia; 4 Australian Research Centre for Human Evolution, Environmental Futures Research Institute, Griffith University, Nathan 4111, Australia; 5 Forensics and Archaeology, School of Environment and Science, Griffith University, Nathan 4111, Australia

**Keywords:** Foresight, planning, bags, slings, organic material culture, long-distance transport, physical offloading, cognitive development

## Abstract

Mobile containers are a keystone human innovation. Ethnographic data indicate that all human groups use containers such as bags, quivers and baskets, ensuring that individuals have important resources at the ready and are prepared for opportunities and threats before they materialize. Although there is speculation surrounding the invention of carrying devices, the current hard archaeological evidence only reaches back some 100,000 years. The dearth of ancient evidence may reflect not only taphonomic processes, but also a lack of attention to these devices. To begin investigating the origins of carrying devices we focus on exploring the basic cognitive processes involved in mobile container use and report an initial study on young children's understanding and deployment of such devices. We gave 3- to 7-year-old children (*N* = 106) the opportunity to spontaneously identify and use a basket to increase their own carrying capacity and thereby obtain more resources in the future. Performance improved linearly with age, as did the likelihood of recognizing that adults use mobile carrying devices to increase carrying capacity. We argue that the evolutionary and developmental origins of mobile containers reflect foundational cognitive processes that enable humans to think about their own limits and compensate for them.

## Graphical abstract

**Figure:**
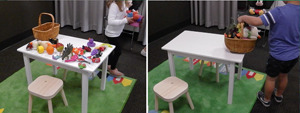

**Media summary:** Mobile containers are a key human innovation. Here we examine what is known about their function, evolution, causation and development.

## Introduction

Humans frequently use mobile containers – such as bags, pockets or slings – to carry resources and tools. So ingrained are they to our present lifeways that their importance in human evolution has been somewhat overlooked (Langley & Suddendorf, 2020). Although there is considerable variation in the materials and techniques employed, as well as in what is commonly carried (see Table 1), a search of the eHRAF World Cultures database found that carrying devices are documented in all hunter–fisher–gatherer communities and other societies across the globe. Why are mobile containers used universally by humans, and what cognitive processes precipitated their invention?

**Table 1.** Recorded uses of mobile containers in hunter–gatherer ethnographies.

Review of the ethnographic literature describing the lifeways of hunter–gatherer communities in Africa, Australia, Asia and the Americas finds the following specific mentions of mobile container use:	
Bathing	Carrying foodstuffs
Carrying equipment (various items)	Carrying infants
Carrying weapons (hunting and/or fighting)	Soaking raw materials
Collection/transport of raw materials for tool-making	Storage of alcohol
Concealment of dangerous objects from sight (inc. ritualized objects)	Storage of beads and body ornaments
Cooking	Storage of colourants
Foodstuff collection/transport (during gathering process)	Storage of drugs and medicines
Infant cradle	Storage of equipment (various tools)
Live animal collection/transport	Storage of foods (short and long term)
Medicinal procedures	Storage of raw materials for tool-making
Mixing of colourants	Storage/transport of water
Mixing of drugs and medicines	Transport of fire
Mixing of drinks, incl. alcohol	Traps
Mixing of resins and glues	Treatment of the deceased
Ritual procedures	Washing items
This list is not exhaustive but is representative of the ethnographic literature available.	

To lay the foundations, we follow Tinbergen (1963) in examining this phenomenon from the four fundamental biological perspectives he called survival value, evolution, causation and ontogeny. Tinbergen's four questions have remained deeply influential in evolutionary reasoning (Bateson & Laland, 2013; Nesse, 2013), especially in providing the critical distinction between *ultimate* (survival value, evolution) and *proximate* (causation, ontogeny) explanations (Scott-Phillips, Dickins & West, 2011). Here we will address each question in turn. We find that very little is currently understood about the ontogeny of the mental capacities involved in mobile container use, and for that reason we present a pilot study that takes some initial steps towards filling out this component of Tinbergen's framework.

We consider a mobile container to be *a tool used for the purpose of holding and transporting things*. In its broadest sense, this definition captures diverse devices that are not prototypically ‘bag-like’, from simple ropes used to tie materials together to modern day containerships that can carry enormous amounts of cargo. Owing to the significant variation in tools that have carrying affordances, we focus our analysis on the shared cognitive requirements that the invention and use of these different artefacts have in common.

## Survival value

The use of mobile containers has obvious functionality in that it allows humans to retain and carry materials across time and space. This ability decreases the likelihood of running out of vital resources, and so can be expected to have fitness benefits. As we will see, advantages for survival and reproduction can also flow from carrying tools that may be useful in particular future situations. Nonetheless, we do not know what pressures brought about the initial use of tools for transportation and safekeeping. Some have speculated that, following the emergence of facultative bipedal ground locomotion, the problem of transportation of infants might have led to the invention of baby slings (Falk, 2009; Taylor, 2010). Other possibilities have to do with the demands of food-gathering (Tanner and Zihlman, 1976) and food-sharing (Isaac, 1971). Although these and other accounts are plausible, there is insufficient evidence to support any one proposal at this time. What is clear, though, is that the emergence of mobile containers must have had considerable downstream effects.

In addition to the immediate increase in carrying capacity for food, drink and sheltering materials, mobile containers created the opportunity to carry and accumulate tools. In particular, our ancestors could keep more tools available than could possibly be carried by two hands alone, which in turn makes investment in the production of additional tools increasingly beneficial. When one is able to retain tools for future use it can become profitable to spend time manufacturing more than what is immediately needed (e.g. where raw materials are available), and to carry tools that are useful only occasionally. The appearance of mobile containers thereby increased the magnitude and benefits of tool innovations and therefore probably played a critical role in driving the emergence of material culture (Langley & Suddendorf, 2020). In a sense, mobile containers are meta-tools, that is, tools that serve as tools for other tools (Matsuzawa, 1994), and so spawned increasing reliance on *toolkits* rather than individual tools.

Today, mobile containers are ubiquitous and incredibly diverse. Our clothes have pockets, we have suitcases for clothes, and trolleys for suitcases. This recursive embedding creates a virtually endless variety of possible combinations. We have even invented large containers that can carry humans themselves, such as boats, cars and planes. Carrying devices are both products and facilitators of our extraordinary cumulative culture, typically filled with artefacts that themselves reflect this material culture. Mobile containers have allowed humans to maintain a local artificial habitat that contains critical resources and the means to fulfil needs, from a knife, to a fire-making kit, to sewing needles. In an important sense, the use of mobile containers has its roots in our capacity to think ahead and to ready ourselves for challenges before they are upon us (Suddendorf, 2006). Indeed, carrying devices enable us to be prepared for all manner of specific future eventualities. This basic capacity for foresight is also probably the key underpinning mechanism, discussed further below, that enabled us to devise these tools in the first place.

## Evolution

Some animals have evolved natural carrying compartments, such as marsupials with pouches to carry offspring and pelicans with throat sacks to carry food. Although it has been argued that non-human primates have only limited use of containers (McGrew, 1992), some animals have on occasion been observed using external containers (see Table 2). However, they do not show much behavioural flexibility or foresight (Suddendorf & Corballis, 1997; Suddendorf, 2013). While many species use tools and some even make them, there is little to suggest any other animals recognize the *future utility* of carrying devices. As we will see in the Causation section, humans can reflect on their own limits and devise ways to physically offload tasks, and then refine them further for more effective long-term use. If these behaviours are uniquely human traits, they are likely to have evolved in hominins over the 6 million years after the split from the line leading to modern chimpanzees.

**Table 2.** Some examples of animal container use

Animal	Container use observed	Reference
African Grey Parrots, Cockatoos	Using nutshells, moss, and a variety of human-made containers, to drink water	Boswall (1983); Pepperberg and Shive (2001); Shumaker et al. (2011)
Ants (some species)	Use pieces of leaf, wood or grass to transport viscous liquids such as honey, fruit pulp or body fluids of prey back to the colony	Banschbach et al. (2006)
Bonobo (in captivity)	Placed a large amount of food on wood-wool, which she then dragged in her enclosure	Walraven et al. (1993)
Capuchin Monkeys, Orangutans, Chimpanzees	Use leaves to scoop up water (chimpanzees in Bossou even fold leaves at about 3 cm intervals between pleats and so they better retain liquid)	Knott (1999); Phillips (1998); Tonooka (2001); van Lawick-Goodall (1968)
Cichlid fish	Lay their eggs on leaf litter and then move the litter when threatened	Keenleyside and Prince (1976)
Chimpanzees (at Gombe)	Sometimes defecate onto leaves while holding them, to then forage for undigested meat amongst the faeces	Goodall (1986)
Chimpanzees (in captivity)	Use coconut shells to collect water	McGrew (1992)
Crow	Transported food with a cup and a Frisbee	Shumaker et al. (2011)
Gorilla (in captivity)	Used a box as a tray, carrying his food around bipedally	Vancatova (2008)
Marsh tit (in captivity)	Detached a paper sticker from its feeder and used the sticky side to pick up powdered food from its bowl and transport it to its perch	Clayton and Jolliffe (1996)
New world Monkeys, Old world monkeys, Great apes	Use of human-made containers (cups, etc.) for drinking water. Some transfer of water over a few metres	Jordan (1982); Parks and Novak (1993); Russon, et al. (2010); Westergaard and Fragaszy (1985)
Orangutan (in captivity)	Filled a tub with straw and transported it outside to make a nest	Shumaker et al. (2011)
Sea otter	Immobilize crabs by wrapping them in kelp and then carry the crabs on their belly while consuming other foods	Riedman and Estes (1990)

The role of containers in human history has become the topic of considerable interest and debate (e.g. Shryock & Smail, 2018). Containers in the form of pottery and baskets have been documented extensively from the late Neolithic (e.g. Nieuwenhuyse, 2019), but their roots are much more ancient. As already noted, there has been speculation that the earliest hominin mobile containers were baby slings (e.g. Bolen, 1992; Ehrenberg, 1989; Falk, 2009; Fisher, 1979), and that containers for gathering would have been useful to Australopithecines over 3 million years ago (Tanner & Zihlman, 1976). However, just because something would have been useful does not mean that it was used. We do not currently know what the original adaptive function was and when it first emerged. Evidence for the transport of raw materials over long distances points back to the Oldowan and Acheulean, but this behaviour only appears to become a regular feature by the second half of the Middle Stone Age (Ambrose, 2012; McBrearty & Brooks, 2000). Even then, it remains possible that transportation could have been achieved without mobile containers. Similarly, because open water appears to have served as a barrier to pre-sapiens populations, evidence for the colonization of islands has been seen to reflect modern cognitive abilities – in particular, that the construction and provisioning of a watercraft (as a mobile container to carry goods and people) required levels of foresight unavailable to archaic hominins (Carter et al., 2019; Ingicco et al., 2018; Leppard, 2015). However, some archaeological discoveries have suggested that such technologies were not necessary to reach islands, and that other processes that did not require advanced planning may have been at work (Brumm et al., 2010; see Leppard 2015 for discussion). The oldest hard evidence for a boat is the Mesolithic ‘Pesse canoe’, found in Assen, Netherlands and dated to about 10,000 years old (Wierenga, 2001).

A recent review of the archeological evidence for mobile containers in the deep past highlights the difficulty of pinpointing the origins and development of these tools. Langley and Suddendorf (2020) found the oldest signs in the archeological record to only date back to about 100,000 years BP. At Blombos (South Africa) and at Qafzeh (Israel) *Homo sapiens* and Neanderthals, respectively, seem to have stored ochre in natural shell containers. If one conceives of necklaces as containers, given that they can contain and transport shells, beads and other objects, then the date can be pushed back further to 130,000 BP, as indicated by white-tailed eagle talons showing cut marks and wear patterns, suggesting that they had been worn by Neanderthals as personal adornment (Radovčić et al., 2015). However, shells or talons appear to be part of the adornment, rather than material to be transported (just as a stone tip can be part of a spear without the stick being considered a mobile container for the stone). The earliest signs of manufactured containers made from stalagmite and wood appear from about 50,000 BP (Carbonell et al., 1996; Cârciumaru et al., 2002, 2015). Evidence for baskets, nets, and pots appears later still, around 30,000 BP, although Hardy et al. (2020) recently reported a small piece of three-ply cord made from inner bark fibres found at a Neanderthal site dated to between 41,000 and 52,000 years BP, which may imply that perishable containers were also present at this antiquity.

The absence of evidence for containers earlier in the archeological record does not necessarily entail evidence for absence (Gamble, 2007). The relatively recent appearance probably reflects the highly perishable nature of the raw materials, and we will need to consider indirect evidence for container construction, for instance, that the 75,000-year-old engraved ochre nodules at Blombos represent instructions for weaving techniques (Anderson, 2020). Langley and Suddendorf (2020) caution that the relatively recent appearance of evidence for mobile containers may also reflect a certain lack of attention to these devices in studies on human evolution. Their association with gathering and ‘women's work’ may have elicited less interest than evidence for hunting (Ehrenberg, 1989; Gero, 1991), and more traces of container technology may be described once this bias is redressed.

## Causation

Given that modern mobile containers range from wallets to backpacks, and from shopping trolleys to cargo containers, the cognitive mechanisms involved in their construction will be diverse. Fundamentally, however, the key first step is the recognition that an object can have a holding and a transporting affordance. For instance, a hollow object such as a gourd or an ostrich egg, or a concave object such as a shell or a tortoise carapace, can be filled and carried. Rather than carrying resources directly in our hands, these objects allow us to ‘offload’ the work of retention to a separate structure often better suited to containing the resource. For the price of having to carry the additional weight of the container, we can be finished with the task of containing – leaving us to only transport the filled container. This trade-off can enable us to carry more material, and often frees our hands for other tasks as well.

We can do much more when we not only recognize immediate utility, but also anticipate the *future* utility of an object. By thinking ahead and considering forthcoming challenges, we can realize what we may need to do now to help us in the future. Recognizing the future utility of solutions is the aspect of foresight that drives us to retain objects that will be useful again, even if they offer nothing in the present moment. It also motivates us to enhance tools, to refine them further so that they become more effective, efficient and reliable. It has been argued that recognition of future utility is essentially what turns a problem solution into an *innovation* (Suddendorf *et al*., 2018; von Hippel & Suddendorf, 2018). Without it, the best solutions to problems would be tossed aside once the task was done. However, with recognition of future utility, we are compelled to retain the object, to protect it, to refine it and possibly to share it with others.

Foresight allows us to anticipate problems, including issues caused by the limits of our physical and mental capacities (Bulley, Redshaw & Suddendorf, 2020). Once we can reflect on the fact that there are limits to our capacities, we can devise ways of compensating for them. Mobile containers enable us to carry great numbers of items more comfortably, after anticipating both (a) the need for objects in the future and (b) one's own inability to carry those objects without an external aid. This *physical offloading* has a parallel to what has come to be known as *cognitive offloading* (Risko & Gilbert, 2016). For instance, because we understand that our memory may let us down in the future, we use external reminders – from knots in handkerchiefs to digital alarms – to ensure that we will remember (Gilbert, 2015; Redshaw et al., 2018b). In this way, humans routinely make thinking easier: taking notes, using calculators and relying on satellite navigation systems. Cognitive offloading enables us to ‘extend’ our mental capacities well beyond their natural limits (Clark & Chalmers, 1998), and is central to modern notions of intelligence (Bocanegra *et al*., 2019). However, physical offloading is probably similarly important for understanding how humans have been able to transform the planet. Tools that extend our physical capacities, from bags to bulldozers, have allowed us to dramatically change our habitats and construct new niches like no other creature could, creating artificial worlds that suit our anticipated needs.

Risko and Gilbert's (2016) influential account of cognitive offloading distinguishes between actions that offload mental processes *onto the body* (e.g. using finger counting) and actions that offload mental processes *into the world* (e.g. using an abacus). A related distinction can be made in the use of mobile carrying devices to transport items, in that a choice is made to offload physical labour onto an external artefact rather than to solely depend on the body. Often, this choice arises from understanding the limits of one's natural carrying capacity, and seeking to expand this limit through artificial means.

## Ontogeny

The development of foresight has become a hot topic in child psychology. Various lines of evidence suggest that children gradually acquire basic capacities over the preschool years (Atance, 2015; Suddendorf, 2017), but that developments continue into late childhood and beyond (Ghetti & Coughlin, 2018; Suddendorf & Redshaw, 2013). Recent studies also suggest that even preschool-aged children can recognize their cognitive limits and compensate for them with *into-the-world* cognitive offloading (Armitage et al., 2020; Bulley et al., 2020). Nonetheless, we are not aware of any studies directly examining children's appreciation of the utility of mobile containers as physical offloading devices, although even young infants may have some understanding of the general concept of containment (Hespos & Baillargeon, 2001), and four-year-olds can secure a tool in a provided container for a return to a problem they had encountered earlier (Redshaw & Suddendorf, 2013). Studying the use of mobile containers offers a new testbed for tracking the early emergence of the capacities involved in anticipating one's own limits and compensating for them. To examine at what point children can reflect on their own physical limits sufficiently to initiate compensatory strategies, we designed the following pilot study of children's understanding and use of mobile containers.

## Methods

### Participants

Participants were recruited from a database of parents who expressed interest in their child participating in research at the Early Cognitive Development Centre of University of Queensland, Australia. The final sample included 106 children between the ages of 3 and 7, who were subdivided into five age categories: 3-year-olds (*n* = 21, *M*_age_ = 40.52 months, SD_age_ = 1.52 months, 10 female), 4-year-olds (*n* = 24, *M*_age_ = 53.65 months, SD_age_ = 2.39 months, 12 female), 5-year-olds (*n* = 22, *M*_age_ = 67.59 months, SD_age_ = 3.46 months, 14 female), 6-year-olds (*n* = 20, *M*_age_ = 76.65 months, SD_age_ = 4.03 months, 8 female) and 7-year-olds (*n* = 19, *M*_age_ = 92.68 months, SD_age_ = 3.79 months, 11 female). A post-hoc power analysis indicated that this sample provided an 88.4% chance of detecting a medium linear effect of age (*r* = 0.30), and a >99.9% chance of detecting a large effect (*r* = 0.50). Age effects of such sizes have been previously observed in studies of children's abilities to consider their future (cognitive) limitations (e.g. Redshaw & Suddendorf, 2016; Redshaw et al., 2018a). Ethical approval was granted by the UQ Human Research Ethics Committee (2018000401).

### Materials

**Figure 1.**
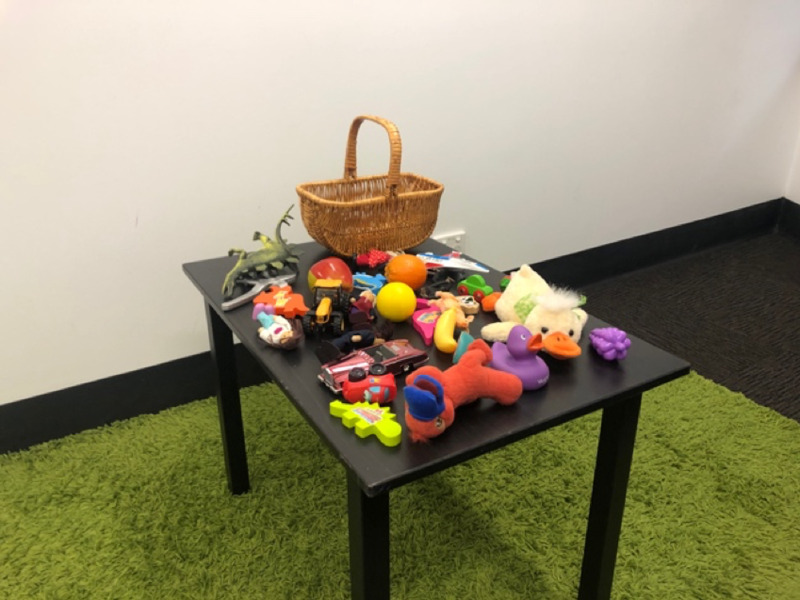
Image of the second experimental room.

The first experimental room contained a stuffed toy tiger named ‘Tigger’. Additionally, this room contained a sheet of stickers that children would later receive as a reward. The second experimental room was located approximately 5 metres’ walk from the first room and contained a stuffed ‘Mickey Mouse’ toy sitting on a chair. This second room also contained a small table with 30 items spread on its surface. Of the items, 29 were various sized toys and one item was a stereotypical Western woven basket (see Figure 1).

### Procedure and measures

The experimenter led children into the first room and pointed out Tigger, before providing the following instructions:

This is Tigger's Room, and he wants to play some games here. But there are no toys here for him to play with. The room next door is Mickey Mouse's room and Mickey has lots of toys. You're going to try bring back as many toys as you can for Tigger. The more toys you bring back, the more stickers you will get from Tigger.

The experimenter then asked children to walk to the second room and pointed out Mickey Mouse, saying: ‘This is Mickey Mouse's room and these are his toys. He wants you to give some of these toys to Tigger’. The experimenter then pointed out five items, with the third item always being the basket (i.e. ‘We have a [toy name], a [toy name], a basket, a [toy name] and a [toy name]’). This was to ensure children were aware the basket was one of the items they could take without prompting them to use it. The experimenter then stated:

We don't have much time before we go back to Tigger's Room. Please take as many toys as you can in one go back to Tigger. Remember, the more toys you take, the more stickers Tigger will give you.

The number of items was large enough to ensure that the children could not carry all of the toys without a carrying device. If the children asked for help, they were told ‘Just take as much as you can in one go’. Additionally, if children asked permission to use the basket, they were told ‘You can do whatever you want to’. The number of items children took (i.e. 0–30) and whether children used the basket (i.e. yes/no) were recorded. Children collected the items and the experimenter showed them back to the first room. The items were given to Tigger and the children were rewarded with stickers.

The children were then asked a series of questions. If they had not used the basket, they were asked, ‘Could you have carried any more toys somehow?’ This measure was coded as (1) yes or (2) no. If children elaborated on how they could have carried toys, their response was coded as (1) using a part of their body, (2) use a mobile-carrying device, or (3) other. Further, children who had not used the basket were asked, ‘Did you see the basket in Mickey's room?’, and the response was coded as (1) yes or (2) no. Regardless of whether children had used the container or not, they were then asked, ‘What do adults do when they have to carry more than they can hold in their hands?’ This variable was coded as (1) use a mobile-carrying device, (2) use the body, (3) social help and (4) no solution.

## Results and discussion

**Figure 2.**
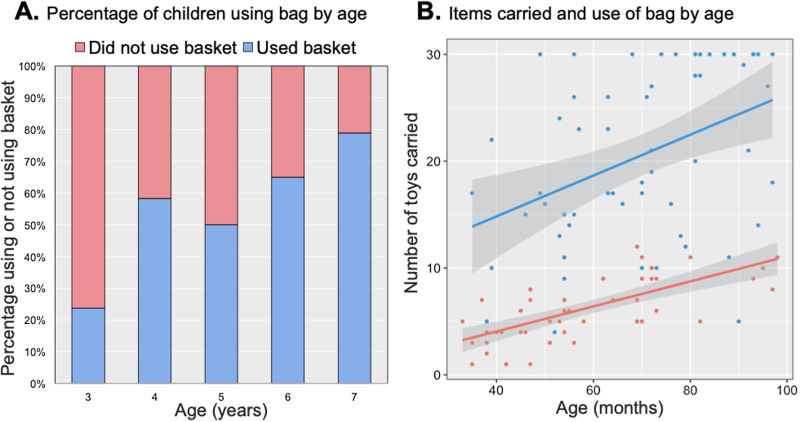
Percentage of children using the bag across age in years (A). There was a linear increase in the use of the bag with age. Number of items carried depending on whether children carried the bag or not across age in months (B). Children carried more items when they used the bag, and performance improved with age.

First, we assessed whether older children used the basket more than younger children. A binomial logistic regression examining the effect of age in months on basket usage was statistically significant, *χ*^2^(1) = 9.81, *p* = 0.002, with increases in age associated with a higher likelihood of children using the basket, *b* = 0.04, SE = 0.01, *p* = 0.003. The model explained 11.8% (Nagelkerke *R*^2^) of the variance in basket usage and correctly classified 61.3% of cases. As shown in Figure 2, the proportion of children using the basket increased roughly linearly with age, from 23.8% of 3-year-olds to 79.0% of 7-year-olds.

Next, we assessed whether older children and those who used the basket carried more items. A multiple linear regression examining the effects of age in months and basket usage on number of items carried was statistically significant (*F*[2,103] = 96.85, *p* < 0.001), with an *R^2^* of 0.653. The number of items carried significantly increased with age, *b* = 0.16, SE = 0.03, *p* < 0.001, and when children used the basket, *b* = 12.56, SE = 1.15, *p* < 0.001.

Descriptive statistics are provided for children's responses to the qualitative questions. Of the 48 children who did not use the basket, 37 (77.1%) claimed that they could have carried more items, with 18 elaborating on how they could do so (nine stated that they could use some part of their body, seven mentioned mobile containers and two provided other responses). Seven (14.6%) of these 48 children claimed that they did not see the basket in Mickey's room despite it being pointed out.

All children (*N* = 106) were asked their perceptions of what adults do when they have too many items: 42.5% of children mentioned a mobile container, 41.5% provided no solution, 11.3% stated adults could get social help and 4.7% said they could use some part of their body. A binomial logistic regression examined the effect of age (in months) on answering this question with some form of a mobile container. The logistic regression model was statistically significant, *χ*^2^(1) = 7.30, *p* = 0.007, with increases in age associated with a higher likelihood of children mentioning a mobile container, *b* = 0.03, *SE* = 0.01, *p* = 0.009. The model explained 8.9% (Nagelkerke *R*^2^) of the variance in mentioning mobile containers and correctly classified 59.4% of cases.

The results of this first study on children's understanding and use of mobile containers suggests that there is considerable early competence. In line with informal observations, young children can use containers to retain and transport materials. Many realize that they can enhance their carrying capacity by using such tools. Yet spontaneous use of a basket to increase their own carrying capacity was only observed in less than a quarter of 3-year-olds. Future research is imperative to examine other facets of early container use and understanding, and its relation to other aspects of cognitive development (Dunbar, 2003; Suddendorf & Redshaw 2013; Stade, 2020). Of particular interest will be examination of how children recognize the future utility of these tools – not just of present utility as examined in this study. It will also be critical to our understanding of the underlying mechanisms to assess the relationship of container use to other measures of foresight.

**Figure 3.**
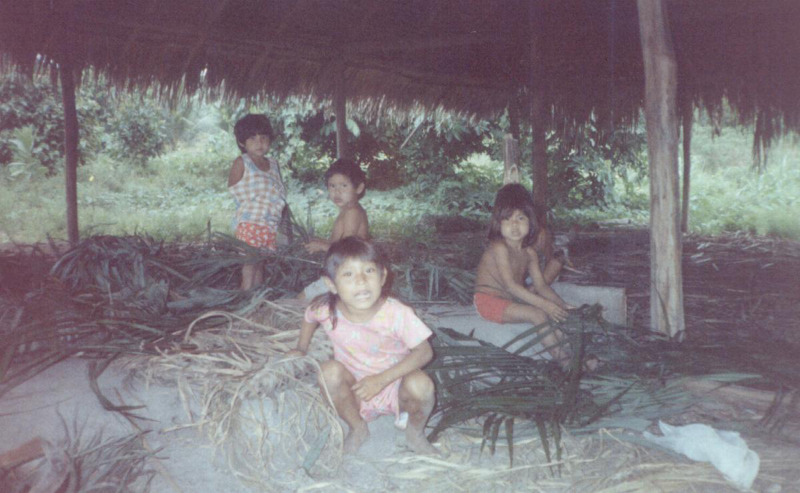
Parakanã children playing at weaving baskets in Brazil (photograph courtesy of Y. Gosso).

Controlled experiments will probably play a large role in building our understanding of this development, as ethnographic data pertaining to children's use of containers is limited. Early ethnographers were largely uninterested in children, and thus, we have only slim insights into their daily activities (Langley & Litster, 2018). There are, however, frequent mentions of children enjoying mimicking the behaviours of their parents (and other adults in their community), which includes making woven baskets (Figure 3; Gosso & Otta 2003) and clay pots (e.g. Crown 2001), among other container types.

## Conclusion

Although non-human animals sometimes use containers, current evidence suggests that only humans recognize the future utility of such devices. Today, mobile containers are all around us. They are so ubiquitous that it is easy to overlook the fundamental importance of this humble innovation. Indicators of manufactured mobile containers in the archeological record appear from around 50,000 years ago, comprising a cord fragment and a couple of wooden and stalagmite containers. Natural containers for ochre processing, such as shells and eggshells, are evident from 100,000 years ago, but this need not entail recognition of future utility or even actual transportation. Yet, we suspect that hominins started relying on mobile containers considerably earlier than either of these dates, perhaps around the time when more tools and especially smaller tools become common in the archeological record.

The emergence of mobile containers in hominin evolution probably drove the innovation of further tools and the ratcheting of ever more sophisticated material culture. With carrying devices our ancestors could obtain a selective advantage by making diverse tools in advance wherever the best resources were. We argue that the appearance of such devices reflects an important milestone in cognitive evolution and development: the capacity to think about one's own limits and compensate for them. This was a game changer. Mobile containers allowed our ancient forebears to maintain a local habitat – to have tools and resources at the ready wherever they went.

In the pilot study, we here demonstrated that even 3-year-old children show some aptitude to use mobile containers to increase carrying capacity and secure immediate benefits. Further studies may examine how children across cultures first begin to recognize the future utility of containers and the items they carry, as well as the role of social learning in this development.

## Research transparency and reproducibility

The data sheets are available at https://osf.io/zadqy/?view_only=3c86f0585ebd44538e794d6c3aeec189
